# Haiku: Philosophy, Metaphysics

**Published:** 2008

**Authors:** Kristina Brenner

Unexpectedly
I slip upon certainty
So I unravel

In the beginning,
Though I can't recall, I'm sure,
Therein lies my end.

I sat by water
As the sun rose before me
A Sandwich of Life

In expectations -
In their very *subversion* -
There lies the sublime.

Take lead from the Fool:
Knowing nothing, nothing hurts.
All is feasible.

What is worse than hate?
An ass, between two hay bales
Starving to its death

Clouded behind words
In time clouds pass, but so too
Does that which they shroud

A star darts away
Failing to see, all the while
It's already caught

Are humans *that* scared
Of a reality that
They cannot cling to?

Tracing the path back
Each day a new regression -
I find my future

